# Photophysical Characterization and *in Vitro* Phototoxicity Evaluation of 5,10,15,20-Tetra(quinolin-2-yl)porphyrin as a Potential Sensitizer for Photodynamic Therapy

**DOI:** 10.3390/molecules21040439

**Published:** 2016-03-31

**Authors:** Letícia D. Costa, Joana de A. e Silva, Sofia M. Fonseca, Cláudia T. Arranja, Ana M. Urbano, Abilio J.F.N. Sobral

**Affiliations:** 1 Molecular Physical Chemistry I & D Unit and Department of Life Sciences, University of Coimbra, Coimbra 3000-456, Portugal; leticiadanielacosta@gmail.com; 2 Department of Chemistry, University of Coimbra, Coimbra 3004-535, Portugal; joana.a.silva@uc.pt (J.A.S.); sfonseca@qui.uc.pt (S.M.F.); claudia.arranja@gmail.com (C.T.A.); 3 Research Centre for Environment, Genetics and Oncobiology (CIMAGO), University of Coimbra, Coimbra 3004-504, Portugal

**Keywords:** photodynamic therapy, photosensitizers, porphyrins, cancer, HT29 cells

## Abstract

Photodynamic therapy (PDT) is a selective and minimally invasive therapeutic approach, involving the combination of a light-sensitive compound, called a photosensitizer (PS), visible light and molecular oxygen. The interaction of these *per se* harmless agents results in the production of reactive species. This triggers a series of cellular events that culminate in the selective destruction of cancer cells, inside which the photosensitizer preferentially accumulates. The search for ideal PDT photosensitizers has been a very active field of research, with a special focus on porphyrins and porphyrin-related macrocycle molecules. The present study describes the photophysical characterization and *in vitro* phototoxicity evaluation of 5,10,15,20-tetra(quinolin-2-yl)porphyrin (2-TQP) as a potential PDT photosensitizer. Molar absorption coefficients were determined from the corresponding absorption spectrum, the fluorescence quantum yield was calculated using 5,10,15,20-tetraphenylporphyrin (TPP) as a standard and the quantum yield of singlet oxygen generation was determined by direct phosphorescence measurements. Toxicity evaluations (in the presence and absence of irradiation) were performed against HT29 colorectal adenocarcinoma cancer cells. The results from this preliminary study show that the hydrophobic 2-TQP fulfills several critical requirements for a good PDT photosensitizer, namely a high quantum yield of singlet oxygen generation (Φ_∆_ 0.62), absence of dark toxicity and significant *in vitro* phototoxicity for concentrations in the micromolar range.

## 1. Introduction

Cancer is one of the leading causes of death worldwide [1]. This disease, characterized by uncontrolled growth and spread of abnormal cells, may be triggered by external or internal factors, such as chemicals, sunlight, hormones, immune conditions and hereditary mutations [2]. Even when effective, the traditional cancer therapies (surgery, radiotherapy and chemotherapy) are associated with long recovery periods and may give rise to serious side effects to the patient [3,4]. On the contrary, photodynamic therapy (PDT), a therapeutic procedure already approved for the treatment of various oncological and non-oncological pathologies, is both minimally toxic and minimally invasive [5].

PDT is based on the specific accumulation of a non-toxic photosensitizer (PS) in the target tissue, followed by local irradiation with visible light at wavelengths matching the absorption spectrum of the photosensitizer [5,6]. Upon light absorption, the photosensitizer is promoted from its singlet ground state (^1^PS) to an unstable excited singlet state (^1^PS*), from which it will decay back to the ground state, either directly or via the excited triplet state (^3^PS*) [5,6]. In the former case, sometimes referred to as the radiative pathway, the excitation energy is discharged through fluorescence, a phenomenon that allows the detection and demarcation of tumors [6,7]. In the latter case, the excited triplet state is reached by intersystem crossing. In this long-lived excited triplet state, the photosensitizer is able to react via one of two competing reactions, commonly described as type I and type II photochemical reactions. Briefly, in the type I reaction, the activated ^3^PS* reacts directly with a biological substrate, via electron or hydrogen transfer, producing free radical species. These species can further react with molecular oxygen (^3^O_2_), giving rise to reactive oxygen species (ROS), such as the superoxide anion, hydrogen peroxide and the hydroxyl radical. In the type II reaction, the activated ^3^PS* transfers its excitation energy directly to molecular oxygen, producing the highly reactive singlet oxygen (^1^O_2_) [5,6,8]. ROS formed via either pathway can oxidize a variety of biomolecules, especially amino acids and unsaturated lipids, compromising their biological functions [4,9,10]. This induces an acute stress response and triggers a series of redox signaling pathways, culminating in cellular death, either directly, by apoptosis and/or necrosis, or indirectly, due to the destruction of the tumor microvasculature, leading to oxygen and nutrient depletion. The inflammation promoted by both mechanisms induces a specific immune response against cancer cells and contributes to the generation of long-term immune memory [11,12]. The contribution of each mechanism is yet to be fully defined, but is known to depend, among other factors, on the nature and intracellular localization of the photosensitizer, as well as on tumor properties [10,11,12].

By allowing local eradication of the disease with minimal side effects, PDT improves the patient’s quality of life and lengthens survival. Moreover, in case of recurrence, the procedure can be repeated safely without harming normal tissues [3,5]. Awareness that the efficiency of this therapeutic approach is critically dependent on the properties of the photosensitizer used prompted, in the last decades, an active search for an ideal photosensitizer, with porphyrins and porphyrin-related macrocycles at the forefront of the photosensitizers developed and clinically used [7,13,14]. This preference can be explained in terms of the selectivity of these compounds towards tumor tissue and of their easy synthesis, versatility, chemical purity and unique photophysical properties (e.g., aromatic stability, high absorption in the red region of the visible spectrum, where light is most penetrating and less harmful to the tissues, and high quantum yields of singlet oxygen generation). Although the mechanisms underlying the preferential accumulation of porphyrin-based photosensitizers in the target cells are still not fully understood, factors relating to tumor architecture and to the chemical properties of the photosensitizer seem to play a critical role. In particular, it is known that the uptake of the photosensitizer by the target tissue is strongly influenced by the hydrophilic-lipophilic balance [15,16].

This work is part of our group’s research effort aimed at developing novel anticancer agents with improved properties [17,18,19,20]. Specifically, we have now explored the suitability for PDT of 5,10,15,20-tetra(quinolin-2-yl)porphyrin (2-TQP), a compound whose crystalline structure was previously solved and published by us [21]. By attaching the quinoline group to the porphyrinic macrocycle, we aimed at adding some of the known medicinal properties of this group (e.g., antitumor, anti-inflammatory, bactericidal and antimalarial) [22] to the stability and favorable photophysical properties generally exhibited by porphyrins. Our results show that 2-TQP exhibits a high quantum yield of singlet oxygen generation and, at the concentrations tested, a significant *in vitro* phototoxicity for concentrations of minimal dark cytotoxicity (against HT29 human colon adenocarcinoma cells), thus fulfilling two critical requirements for a good PDT photosensitizer.

## 2. Results and Discussion

### 2.1. Photophysical Charaterization

**Figure 1:**
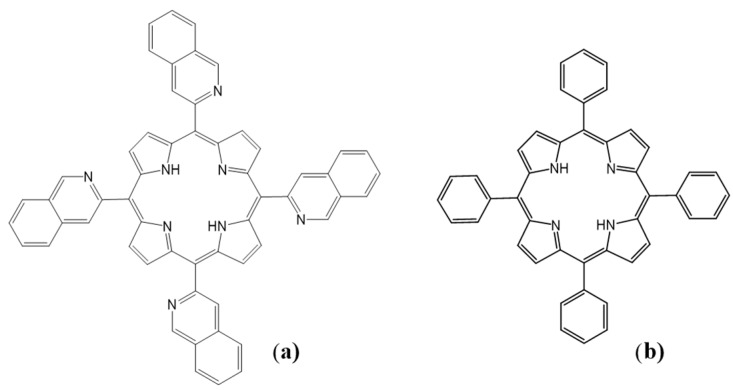
Structural representation of (**a**) 2-TQP and (**b**) TPP.

**Figure 2:**
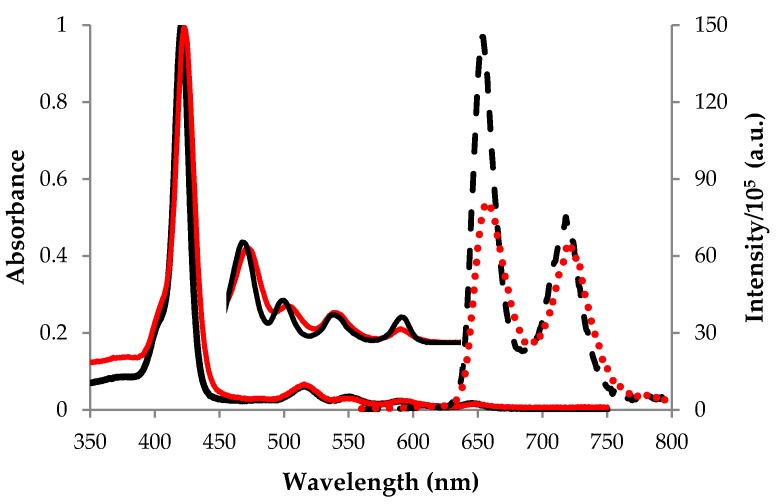
Absorption (–) and fluorescence emission (···) spectra of 2-TQP (red) and TPP (black) recorded at room temperature in tetrahydrofuran (THF) and deaerated toluene solutions, respectively. Absorbance was monitored from 350 to 750 nm and fluorescence emission upon excitation at 552 nm was monitored from 560 to 800 nm.

The structure of the 2-TQP porphyrin characterized in this study is shown in Figure 1a. 2‑TQP is a free-base porphyrin that has a typical *ethio*-type UV-Vis spectrum, composed of an intense Soret band and four less intense Q bands (Figure 2). Table 1 lists the molar absorption coefficients (ε) for these five bands, which were calculated using the Beer-Lambert law. The quality of the linear fits (*R*^2^ ≥ 0.998) indicates that no aggregation occurred in solution under the experimental conditions employed for these determinations.

Figure 2 shows also the steady-state fluorescence emission spectrum of 2-TQP, as well as that of the reference 5,10,15,20-tetraphenylporphyrin (TPP, Figure 1b), upon excitation at 552 nm. The calculated fluorescence quantum yield (Ф_F_) of 2-TQP in toluene (0.095; Table 1) indicates that its excitation energy is not excessively lost by the radiative pathway, an important feature in the context of PDT [6].

Most porphyrin-based compounds are type II photosensitizers, meaning that their photodynamic activity depends on the formation of the highly reactive singlet oxygen [23]. As such, the potential of these compounds as PDT photosensitizers depends critically on their quantum yield of singlet oxygen generation (Ф_∆_), a parameter that reflects the number of ^1^O_2_ molecules generated per number of photons absorbed by the sensitizer [9,14]. In this work, singlet oxygen phosphorescence (^3^Σ_g_ → ^1^Δ_g_) was directly measured at 1270 nm, after laser excitation at 355 nm.

**Figure 3:**
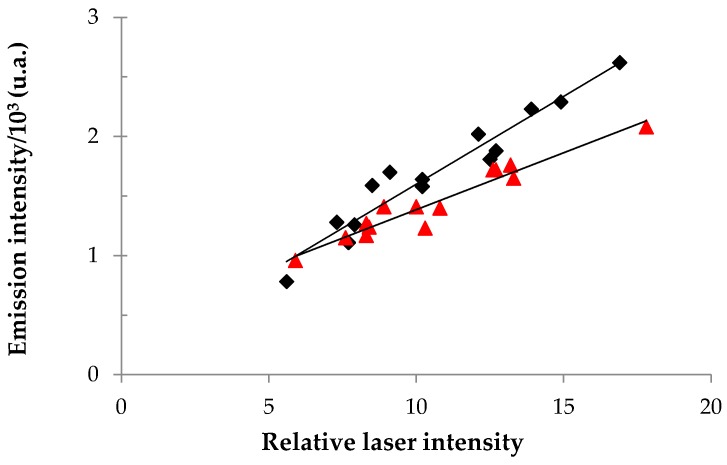
Plots of singlet oxygen emission intensity at 1270 nm as a function of relative laser intensity, for 2-TQP (red) and for 1*H*-phenalen-1-one (black). Measurements were carried out in tetrahydrofuran solutions. Linear regressions gave the following equations: *y* = 9.55 × 10^−5^*x* + 4.30 × 10^−4^ (*R*^2^ = 0.931), for 2-TQP, and *y* = 1.47 × 10^−4^*x* + 1.25 × 10^−4^ (*R*^2^ = 0.933), for 1*H*-phenalen-1-one.

More specifically, plots of ^1^O_2_ phosphorescence intensity at 1270 nm as a function of the laser power were produced for both 2-TQP and for 1*H*-phenalen-1-one (Figure 3), an aromatic ketone that is commonly used as a reference in the determination of singlet oxygen quantum yields [24]. The Φ_∆_ value for 2-TQP was calculated from the ratio of the slopes of these two plots, considering a Φ_Δ_ value of 0.95 for 1*H*-phenalen-1-one [24].

Our results show that 2-TQP has a high quantum yield of singlet oxygen generation (0.62 in tetrahydrofuran). Significantly, the calculated value is above the range reported for most of the photosensitizers employed in PDT (*i.e.*, 0.3–0.5) [8,10], including Photofrin^®^ in toluene (Φ_∆_ = 0.32) [25] and Foscan^®^ in methanol (Φ_∆_ = 0.43) [26], which is a good promise for the use of 2-TQP as a PDT photosensitizer.

### 2.2. In Vitro Cytotoxicity: Preliminary Evaluation

As 2-TQP is a hydrophobic porphyrin, with a minLog *P* value higher than 9 (see Table S1 in the Supplementary Material), the concentrated solution used in the cytotoxicity assays was prepared in an organic solvent, dimethylsulfoxide (DMSO). Treatments were carried out with a 1 mg/mL solution, which was the highest concentration achieved in this solvent. For each condition tested, cell viability was expressed as a percentage of the viability of the corresponding control culture, grown in the absence of 2-TQP, but exposed to the same DMSO concentration as all 2-TQP-treated cultures, *i.e.*, 0.5% (*v*/*v*). This DMSO concentration was not overly cytotoxic to HT29 cultures, as previously evaluated by us [17].

**Figure 4:**
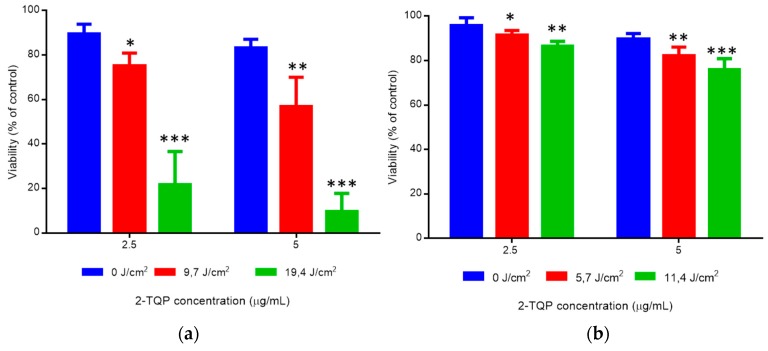
The photodynamic activity of 2-TQP against HT29 cell cultures was determined by the MTT assay, as described in the Experimental Section. Briefly, 24 h after 2-TQP addition, cultures were irradiated for 15 or 30 min (**a**) in the absence (fluence rate of 10.8 mW/cm^2^) or (**b**) in the presence of an OG570 longpass filter (fluence rate of 6.3 mW/cm^2^). For 2-TQP, 2.5 and 5.0 μg/mL correspond to 3.1 and 6.1 μM, respectively. The corresponding non-irradiated cultures were established and processed in parallel. Viability was determined 24 h after irradiation and expressed as a percentage of the viability of the corresponding control cultures, grown in the absence of 2-TQP. Data represent the mean ± SD (*n* ≥ 5). * *p* < 0.05, ** *p* < 0.01 and *** *p* < 0.001, when compared with the corresponding non-irradiated cultures, with Two-Way ANOVA followed by Dunnett’s multiple comparison test.

The results obtained in our evaluation of the photodynamic activity of 2-TQP against HT29 cells can be seen in Figure 4. Cultures were irradiated with a slide projector equipped with a halogen lamp, both in the absence (Figure 4a) and in the presence of an optical longpass orange glass filter (Figure 4b). In both cases, the photodynamic activity of 2-TQP depended both on porphyrin concentration and light dose. As expected, a much higher photodynamic activity was achieved in the absence of the longpass filter. In fact, by filtering all radiation with wavelengths shorter than 570 nm, the longpass filter employed prevented excitation of the Soret band of the photosensitizer, where it absorbs *ca.* 30 to 100 times more than in the 590 and 648 nm Q bands (Table 1). Nonetheless, even in the presence of the filter, 2-TQP still produced statistically significant decreases in cell viability, whilst exhibiting minimal dark toxicity, as required for an efficient PDT photosensitizer.

It might be argued that the results obtained in the presence of the longpass filter are more relevant in the context of PDT than those obtained in its absence. In fact, as the light used to excite the Soret band has low tissue penetration (*ca.* 1 mm), it can only produce superficial tissue necrosis, and is not useful for the treatment of deep and/or large tumors [5]. Still, it is successfully used for the treatment of dermatological lesions, including actinic keratosis [27]. Also, the results obtained with non-filtered white light are also relevant for comparison purposes, as many studies reported in the literature employed a similar light [23,28,29]. However, comparisons between our results and those obtained in other *in vitro* studies assessing other photosensitizers are not straightforward, as the experimental conditions invariably differ in terms of other parameters affecting the end result, namely in terms of cell line, incubation time, light source and light dose. Using a protocol very similar to ours, including the use of filtered light and a light dose of 4 J/cm^2^, but a different cell line (the murine colon carcinoma Colo-26 cell line), You and co-workers achieved a 50% decrease in cell viability by exposing cultures to 10 µM of Photofrin^®^ [30]. Due to the limited water solubility of 2-TQP, it was not possible to expose cells to concentrations higher than 5 μg/mL (6.1 μM), which prevented the calculation of exact IC_50_ values. However, it is worth mentioning that the IC_50_ values calculated, by extrapolation, using the linear regression equations of the viability *versus* concentration curves (17 µM, for a light dose of 5.7 J/cm^2^ (*R*^2^ = 0.9986), and 13 µM for a light dose of 11.4 J/cm^2^ (*R*^2^ = 0.9969)) compare well with the above-mentioned value for Photofrin^®^. In another study, the photodynamic effect of Foscan^®^, whose miLog *P* value is only slightly lower than that of 2-TQP (Table S1), was evaluated in the cell line used by us. Upon irradiation of HT29 cell cultures with an Ar+ laser at 504 nm with a light dose of 5 J/cm^2^, Laville and co-workers observed a decrease in cell viability of around 80% upon exposure to 2 µM of Foscan^®^ and a decrease of 95% upon exposure to 8 µM [31]. In a more recent study published by the same group, also with HT29 cells, this time irradiated with red light (>540 nm, 1.8 J/cm^2^), the IC_50_ determined after 24 h of incubation was 0.8 µM) [32]. However, comparisons with our results are not straightforward, as in our protocol cell viability was determined 24 h after irradiation, whereas in their protocol the viability was determined only after 72 h, which might have contributed to the very low IC_50_ value.

The hydrophobic nature of 2-TQP not only imposes limits on the concentrations that can be achieved in aqueous milieu in its free form, it may also translate into some aggregation in this milieu, which might compromise some of its key photophysical properties, in particular its ability to absorb light and generate singlet oxygen [7,14,33]. On the other hand, it might favor its accumulation in biological membranes, namely at the level of the cytoplasmic, mitochondrial and lysosomal membranes [33,34]. In fact, almost all FDA approved PDT agents are hydrophobic [34]. Thus, the development of efficient targeted carriers to improve the selective accumulation of hydrophobic photosensitizers is clearly one of the main challenges in PDT. This is evidenced by the increasing number of published studies addressing the selective delivery of photosensitizers to target cells [34,35]. Among the numerous vehicles used to improve tumor accumulation are organic and inorganic nanoparticles, liposomes, cyclodextrins, natural or synthetic biocompatible polymers (chitosan, micelles, dendrimers, hydrogels, among others), carbon nanomaterials such as fullerene or carbon nanotubes and the quantum dots. Although the use of carriers has proved efficient, the direct conjugation of the PS with biomolecules with high selectivity towards tumor-associated molecular targets (e.g., overexpressed cell surface receptors that are not usually expressed in healthy cells), namely conjugation with antibodies against tumor antigens, sugars, hormones or receptors, seems to be more promising [36]. These targeted delivery strategies confer a greater solubility to the hydrophobic photosensitizers, preventing their aggregation in the blood stream, thus enhancing their accumulation in the target tissue, while reducing the toxicity against normal tissues [35].

## 3. Experimental Section

### 3.1. Materials

**Scheme 1:**
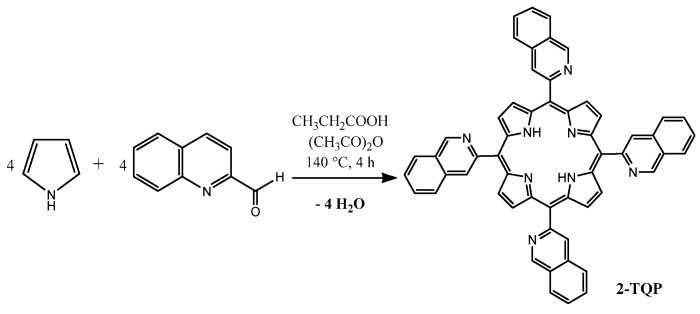
Synthesis of 2-TQP (5,10,15,20-tetra(quinolin-2-yl)porphyrin).

With the exception of pyrrole, which was distilled under reduced pressure prior to use, all reagents employed in porphyrin synthesis were used as supplied by Sigma-Aldrich Química (Sintra, Portugal). The culture medium (RPMI-1640), DMSO and 3-(4,5-dimethylthiazol-2-yl)-2,5-diphenyl-tetrazolium bromide (MTT) were also obtained from Sigma-Aldrich Química. Gibco^®^ fetal calf serum was obtained from Thermo Fisher Scientific Inc. (Waltham, MA, USA). 2-TQP (Figure 1a) was synthesized by condensation of 2-quinolinecarboxaldehyde with pyrrole in acid media, according to a procedure already described by us [21] (Scheme 1).

Physical data obtained for 2-TQP (C_56_H_34_N_8_): Yield: 5% (76 mg). ^1^H-NMR (400 MHz, CDCl_3_/TMS), δ (ppm)): −2.58 (s, 2H, NH); δ 7.8 (t, 4H, 2-quin.); δ 7.95 (t, 4H, 2-quin.); δ 8.15 (d, 4H, 2-quin.); δ 8.41 (d, 4H, 2-quin.); δ 8.45 (d, 4H, 2-quin.); δ 8.52 (d, 4H, 2-quin.); δ 8.88 (s, 8H, Hβ-pyrrol). HPLC (rt = 8.58–8.72 min). MS-ESI(+): *m*/*z* calculated for C_56_H_34_N_8_ = 818.29, found 819.8 [M + H]^+^. UV-Vis (THF): λ_max_ (ε (M^−1^cm^−1^), R^2^) 422.5 nm (1.24 × 10^5^, 0.998), 515.5 (9.92 × 10^3^, 0.999); 552.0 (4.54 × 10^3^, 0.999); 590.0 (3.39 × 10^3^, 0.998); 648.0 (1.85 × 10^3^, 0.998). TPP (CAS [917-23-7]) was synthesized from pyrrole and benzaldehyde, following the procedure described by Adler *et al.* [37,38]. Spectroscopic data were in full agreement with those already published.

### 3.2. Chemical Characterization

Absorption spectra (UV-Vis) were recorded with an USB4000 fiber optic spectrophotometer (Ocean Optics, Dunedin, FL, USA) using THF as solvent and a 1 cm path length quartz cell. ^1^H-NMR spectra were recorded on an Avance III 400 MHz spectrometer (Bruker, Billerica, MA, USA) using CDCl_3_ as the solvent and tetramethylsilane (TMS) as internal standard. All spectra were recorded at room temperature. The ^1^H-NMR spectrum of 2-TQP is presented in Figure S1. High performance liquid chromatography-mass spectrometry (HPLC-MS) studies were conducted using an Ultimate 3000 system (Dionex, Sunnyvale, CA, USA) coupled to a Bruker AmaZon SL mass spectrometer equipped with an electrospray ionization (ESI) source, operating in positive mode. Liquid chromatography was carried out with a reverse phase Hichrom 5 C_18_ (150 × 4.6 mm) column, using a gradient of acetonitrile and formic acid/water (0.1% (*v*/*v*) of formic acid), programmed as follows: 0–5 min, 60% acetonitrile; 5–7 min, 70% acetonitrile; 7–10 min, 80% acetonitrile; 10–20 min, 90% acetonitrile and 100% acetonitrile afterwards. The mobile phase was delivered at a flow rate of 0.80 mL/min. The HPLC chromatogram as well as the MS and MS/MS spectra obtained are shown in Figures S2 and S3, respectively.

### 3.3. Photophysical Characterization

#### 3.3.1. Determination of Molar Absorption Coefficients

Molar absorption coefficients were determined by direct application of the Beer-Lambert law, using solutions of 2-TQP in THF with concentrations ranging from 10^−6^ to 10^−5^ M.

#### 3.3.2. Determination of Fluorescence Quantum Yield

Fluorescence spectra of 2-TQP and TPP in toluene solutions, deaerated by bubbling with nitrogen, were recorded at room temperature with a Spex Fluorolog 3–22 spectrophotometer (Horiba-Jobin-Yvon Inc., Edison, NJ, USA) using quartz cells. These spectra were corrected for the wavelength response of the system. The fluorescence quantum yield (Φ_F_) of 2‑TQP was then calculated by comparing the area below its corrected emission spectrum with the area below the corresponding spectrum of TPP, which was used as a fluorescence standard (Φ_F_ = 0.11) [39].

#### 3.3.3. Determination of Singlet Oxygen Quantum Yield

The singlet oxygen quantum yield (Φ_F_) of 2-TQP was determined by direct measurement of the phosphorescence at 1270 nm, using an adaptation of the LKS.60 flash kinetic spectrometer (Applied Photophysics, Leatherhead, UK). The modification involved the interposition of a dielectric mirror (08MLQ005/345, Melles Griot, Albuquerque, NM, USA) that reflects more than 99.5% of the incident light in the 610–860 nm range, and a RG665 red and black longpass filter (Schott AG, Mainz, Germany). In addition, a 600 line diffraction grating was mounted in place of the standard one to extend spectral response to the infrared. The filter employed is essential in eliminating from the infrared signal all the first harmonic contributions of the sensitizer emission in the 500–800 nm range [17].

Singlet oxygen emissions of aerated solutions of 2-TQP and of the reference sensitizer 1*H*-phenalen-1-one, in THF and contained in quartz cells, were detected, at room temperature, using a R5509-42 photomultiplier (Hamamatsu Photonics K. K., Hamamatsu City, Japan) cooled to 193 K in a liquid nitrogen chamber (model PC176TSCE005), following excitation by the third harmonic (355 nm) of a Nd:YAG (Quanta Ray GCR 130, Spectra-Physics, Santa Clara, CA, USA) laser. The sample concentrations were chosen to yield absorbance readings of 0.30–0.35 at the excitation wavelength. The singlet oxygen quantum yield of 2-TQP was calculated by comparing its singlet oxygen intensity emission with that obtained for the reference sensitizer, whose Φ_∆_ = 0.95 [24].

### 3.4. Dark Cytotoxicity and Phototoxicity Evaluations

#### 3.4.1. Cell Culture

All cytotoxicity evaluations were performed in the human colorectal adenocarcinoma cell line HT29, purchased from the American Type Culture Collection (No. HTB-38, ATCC, Teddington, Middlesex, UK). HT29 cells were grown as monolayers in RPMI-1640 medium supplemented with 10% (*v*/*v*) heat inactivated fetal bovine serum, in a humidified atmosphere of 95% air and 5% CO_2_, at 37 °C.

#### 3.4.2. Porphyrin Treatment

For the evaluation of dark cytotoxicity and phototoxicity, cultures were prepared in 24-well plates at a seeding density of 3 × 10^4^ cells/cm^2^, in 1 mL of RPMI-1640 culture medium. Final porphyrin concentrations in the culture medium of 2.5 and 5 µg/mL were achieved by adding to the culture medium an adequate volume of a 1 mg/mL solution of 2-TQP in dimethylsulfoxide (DMSO) ≥ 99.9%. This porphyrin solution was filter-sterilized and kept in the dark to avoid photobleaching. For each experiment, control cultures, treated with DMSO only, were established and processed in parallel. For all cultures, the final DMSO concentration was always 0.5% (*v*/*v*) and all treatments were carried out 24 h after seeding. Quadruplicate cultures were prepared for each experimental condition.

#### 3.4.3. Photodynamic Treatment

After porphyrin and/or vehicle addition, porphyrin-treated and control cultures were incubated for 24 h under standard conditions. The culture medium was then carefully removed and the monolayers were rinsed three times with a phosphate-buffered saline solution (PBS). All non-internalized 2-TQP (as well as all DMSO present in the growth media) was thus removed. Afterwards, each culture received 1 mL of a PBS solution supplemented with 10 mM of d-glucose. Replacement of the culture medium by this solution ensured the absence of constituents, such as riboflavin, that could act as potent photosensitizers, thereby masking the photodynamic activity of 2-TQP [40,41]. Cell cultures were then exposed to different light doses ranging from 0 (dark controls) to 19.4 J/cm^2^. Immediately after irradiation, the glucose-containing PBS solution was replaced by fresh RPMI-1640 medium and cultures were incubated for a further 24 h in the dark. Their viability was then assessed as described below (Section 3.4.5). Non-treated cultures were subjected to the same irradiation regimens as the corresponding treated cultures and dark controls were treated with the same porphyrin concentrations as the corresponding irradiated cultures, but were kept in the dark for the whole duration of the irradiation procedure and under the same environmental conditions as the irradiated cultures. For each independent experiment, all cultures were established and processed in parallel.

#### 3.4.4. Light Source

Cultures were irradiated with a Reflecta Diamator slide projector (AGFA−Gevaert N.V., Mortsel, Belgium) equipped with a 150 W low voltage halogen lamp (Xenophot HLX 64640, OSRAM, Munich, Germany), either in the absence or in the presence of an optical longpass orange glass filter (OG570, Schott AG, Mainz, Germany) that only allowed the transmission of radiation with wavelengths longer than 570 nm. The experiments were performed with a fluence rate of either 10.8 mW/cm^2^ (in the absence of the longpass filter) or 6.3 mW/cm^2^ (in the presence of the longpass filter), as measured with a 212 power meter (Coherent Inc., Santa Clara, CA, USA). Light was delivered for 15 or 30 min and filtered through a water layer of *ca.* 1 cm for heat absorption.

#### 3.4.5. Cytotoxicity Evaluations

Cytotoxicity was assessed in terms of dehydrogenase activity, using the MTT viability assay. This method is based on the reduction of the MTT salt by metabolically active (*i.e.,* viable) cells, into a purple colored formazan product, whose absorbance at 570 nm is proportional to the number of viable cells [42]. For this purpose, 24 h after irradiation, 150 µL of a 5 mg/mL MTT solution in PBS were added to each well, and cultures were incubated for a further 4 h at 37 °C. The resulting formazan crystals were dissolved by the addition of 300 µL of DMSO. Optical densities were measured at 570 nm in a FLx800 microplate reader (BioTek Instruments Inc., Winooski, VT, USA).

#### 3.4.6. LogP *in Silico* Calculations

The miLog*P* was calculated using Molinspiration WebME Editor 3.81. The parameters for drug-likeness were evaluated according to the Lipinski’s “rule-of-five”, using the Molinspiration WebME Editor (http://www.molinspiration.com). These parameters are presented in Table S1 of the Supplementary Materials.

#### 3.4.7. Statistical Analysis

GraphPad Prism (version 5.00, GraphPad Software, San Diego, CA, USA) was used to perform the statistical analysis. The statistical comparisons between the treated and control groups were performed using ANOVA followed by Dunnett’s multiple comparison *post hoc* test. Results are expressed as the mean ± standard deviation. The level of significance was set at *p* < 0.05.

## 4. Conclusions

In this manuscript, we describe the photophysical characterization and the photodynamic effect against the colon adenocarcinoma cell line HT29 of 2-TQP porphyrin. Altogether, our results show that 2-TQP meets several essential requirements of an ideal photosensitizer: it is a pure compound of known chemical composition, has good absorbance in the visible region of the spectrum, including the red spectral region (λ_max_ = 648, nm in this region) and produces singlet oxygen efficiently. Furthermore, it is harmless to these cells in the absence of light, becoming cytotoxic when activated by light, presumably due to the generation of singlet oxygen. Of note, the photodynamic effect observed in this preliminary evaluation was concentration-dependent. Thus, we anticipate that more pronounced effects will be produced using higher concentrations, which can be achieved through the use of a suitable carrier for this hydrophobic porphyrin. By preventing porphyrin aggregation, the carrier should also promote a greater accumulation in the target cells and, ultimately, an even higher photodynamic effect.

## Figures and Tables

**Table 1:** Photophysical properties of 2-TQP at room temperature ^a^.

λ_max,abs_ (nm) ε (M^−1^·cm^−1^) ^a^					λ_max,em_ (nm)		Ф_F_^a^	Ф_Δ_
Soret	Qy_(1–0)_	Qy_(0–0)_	Qx_(1–0)_	Qx_(0–0)_	Q_(0–0)_	Q_(0–1)_		
422.5 1.24 × 10^5^	515.5 9.82 × 10^3^	552.0 4.54 × 10^3^	590.0 3.39 × 10^3^	648.0 1.85 × 10^3^	657	722	0.095	0.62

^a^ Absorption values were determined in tetrahydrofuran and fluorescence values were determined in toluene. For a complete description of the methods used, see the Experimental Section; λ_max,abs_: wavelength of maximum absorption; ε: Molar absorption coefficient; λ_max,em_: Peak fluorescence wavelength; Ф_F_: Fluorescence quantum yield; Ф_Δ_: Singlet oxygen quantum yield.

## Supplementary Materials

Supplementary materials can be accessed at: http://www.mdpi.com/1420-3049/21/4/439/s1.

## Abbreviations

The following abbreviations were used in this manuscript:

DMSO: Dimethylsulfoxide
ESI: Electrospray ionization
HPLC-MS: High performance liquid chromatography-Mass spectrometry
MTT: 3-(4,5-Dimethylthiazol-2-yl)-2,5-diphenyltetrazolium bromide
NMR: Nuclear magnetic resonance
PDT: Photodynamic therapy
PBS: Phosphate-buffered saline
PS: Photosensitizer
ROS: Reactive oxygen species
THF: Tetrahydrofuran
2-TQP: 5,10,15,20-tetra(quinolin-2-yl)porphyrin
TPP: 5,10,15,20-tetraphenylporphyrin

## References

[1] Siegel R., Ma J., Zou Z., Jemal A. (2014). Cancer statistics. CA Cancer J. Clin..

[2] American Cancer Society (2015). Cancer Facts & Figures.

[3] Triesscheijn M., Baas P., Schellens J.H., Stewart F.A. (2006). Photodynamic therapy in oncology. Oncologist.

[4] Kudinova N.V., Berezov T.T. (2010). Photodynamic therapy of cancer: Search for ideal photosensitizer. Biochem. (Mosc) Suppl. Ser. B Biomed. Chem..

[5] Agostinis P., Berg K., Cengel K.A., Foster T.H., Girotti A.W., Gollnick S.O., Hahn S.M., Hamblin M.R., Juzeniene A., Kessel D. (2011). Photodynamic therapy of cancer: An update. CA Cancer J. Clin..

[6] Castano A.P., Demidova T.N., Hamblin M.R. (2004). Mechanisms in photodynamic therapy: Photosensitizers, photochemistry and cellular localization. Photodiagn. Photodyn..

[7] Ethirajan M., Chen Y., Joshi P., Pandey R.K. (2011). The role of porphyrin chemistry in tumor imaging and photodynamic therapy. Chem. Soc. Rev..

[8] Plaetzer K., Krammer B., Berlanda J., Berr F., Kiesslich T. (2009). Photophysics and photochemistry of photodynamic therapy: Fundamental aspects. Lasers Med. Sci..

[9] DeRosa M.C., Crutchley R.J. (2002). Photosensitized singlet oxygen and its applications. Coord. Chem. Rev..

[10] Juzeniene A., Nielsen K.P., Moan J. (2006). Biophysical aspects of photodynamic therapy. J. Environ. Pathol. Toxicol. Oncol..

[11] Allison R.R., Moghissi K. (2013). Photodynamic therapy (PDT): PDT mechanisms. Clin. Endosc..

[12] Robertson C.A., Hawkins D., Abrahamse E.H. (2009). Photodynamic therapy (PDT): a short review on cellular mechanisms and cancer research applications for PDT. J. Photochem. Photobiol. B Biol..

[13] Vicente M.G.H. (2001). Porphyrin-based sensitizers in the detection and treatment of cancer: Recent progress. Curr. Med. Chem. Anti-Cancer Agents.

[14] Nyman E.S., Hynninen P.A. (2004). Research advances in the use of tetrapyrrolic photosensitizers for photodynamic therapy. J. Photochem. Photobiol. B Biol..

[15] Solban N., Rizvi I., Hasan T. (2006). Targeted photodynamic therapy. Lasers Surg. Med..

[16] Osterloh J., Vicente M.G.H. (2002). Mechanisms of porphyrinoid localization in tumors. J. Porphyr. Phthalocyanines.

[17] Silva P., Fonseca S.M., Arranja C.T., Burrows H.D., Urbano A.M., Sobral A.J.F.N. (2010). A new nonconjugated naphthalene derivative of *meso*-*tetra*-(3-hydroxy)-phenyl-porphyrin as a potential sensitizer for photodynamic therapy. Photochem. Photobiol..

[18] Silva-Carvalho R., Gonçalves V.M., Ferreira A.M., Cardoso S.M., Sobral A.J.F.N., Almeida-Aguiar C., Baltazar F. (2014). Antitumoural and antiangiogenic activity of portuguese propolis in *in vitro* and *in vivo* models. J. Funct. Foods.

[19] Santos A., Rodrigues A.M., Sobral A.J.F.N., Monsanto P.V., Vaz W.L., Moreno M.J. (2009). Early events in photodynamic therapy: Chemical and physical changes in a POPC:cholesterol bilayer due to photosensitization of hematoporphyrin IX. Phothochem. Phothobiol..

[20] Sobral A.J.F.N., Eléouet S., Rousset N., Gonsalves A.M.d'A.R., Le Meur O., Bourré L., Patrice T. (2002). New sulphonamide and sulphonic ester porphyrins as sensitizers for photodynamic therapy. J. Porphyr. Phthalocyanines.

[21] e Silva J.D.A., Domingos V.F., Marto D., Costa L.D., Marcos M., Silva M.R., Sobral A.J.F.N. (2013). Reversible sequestering of CO_2_ on a multiporous crystalline framework of 2-quinolyl-porphyrin. Tetrahedron Lett..

[22] Graves P.R., Kwiek J.J., Fadden P., Ray R., Hardeman K., Coley A.M., Foley M., Haystead T.A.J. (2002). Discovery of novel targets of quinoline drugs in the human purine binding proteome. Mol. Pharmacol..

[23] Deda D.K., Pavani C., Caritá E., Baptista M.S., Toma H.E., Araki K. (2012). Correlation of photodynamic activity and singlet oxygen quantum yields in two series of hydrophobic monocationic porphyrins. J. Porphyr. Phthalocyanines.

[24] Schmidt R., Tanielian C., Dunsbach R., Wolff C. (1994). Phenalenone, a universal reference compound for the determination of quantum yields of singlet oxygen O_2_(^1^Δ_g_) sensitization. J. Photochem. Photobiol. A Chem..

[25] Redmond R.W., Gamlin J.N. (1999). A compilation of singlet oxygen yields from biologically relevant molecules. Photochem. Photobiol..

[26] Bonnett R., Charlesworth P., Djelal B.D., Foley S., McGarveyb D.J., Truscott T.G. (1999). Photophysical properties of 5,10,15,20-tetrakis(m-hydroxyphenyl)-porphyrin (m-THPP), 5,10,15,20-tetrakis(m-hydroxyphenyl) chlorin (m-THPC) and 5,10,15,20-tetrakis(m-hydroxyphenyl)bacteriochlorin (m-THPBC): A comparative study. J. Chem. Soc. Perkin Trans..

[27] Wiegell S.R., Wulf H.C., Szeimies R.M., Basset-Seguin N., Bissonnette R., Gerritsen M.J., Gilaberte Y., Calzavara-Pinton P., Morton C.A., Sidoroff A. (2012). Daylight photodynamic therapy for actinic keratosis: An international consensus. J. Eur. Acad. Dermatol. Venereol..

[28] Silva E.M., Giuntini F., Faustino M.A., Tomé J.P., Neves M.G., Tomé A.C., Silva A.M., Santana-Marques M.G., Ferrer-Correia A.J., Cavaleiro J.A. (2005). Synthesis of cationic β-vinyl substituted meso-tetraphenyl porphyrins and their *in vitro* activity against herpes simplex virus type 1. Bioorg. Med. Chem. Lett..

[29] Hatakeyama T., Murayama Y., Komatsu S., Shiozaki A., Kuriu Y., Ikoma H., Nakanishi M., Ichikawa D., Fujiwara H., Okamoto K. (2013). Efficacy of 5-aminolevulinic acid-mediated photodynamic therapy using light-emitting diodes in human colon cancer cells. Oncol. Rep..

[30] You Y., Gibson S.L., Hilf R., Davies S.R., Oseroff A.R., Roy I., Ohulchanskyy T.Y., Bergey E.J., Detty M.R. (2003). Water soluble, core-modified porphyrins. 3. Synthesis, photophysical properties, and *in vitro* studies of photosensitization, uptake, and localization with carboxylic acid-substituted derivatives. J. Med. Chem..

[31] Laville I., Figueiredo T., Loock B., Pigaglio S., Maillard P.H., Grierson D.S., Carrez D., Croisy A., Blais J. (2003). Synthesis, cellular internalization and photodynamic activity of glucoconjugated derivatives of tri and tetra(meta-hydroxyphenyl)chlorins. Bioorg. Med. Chem..

[32] Maillard Ph., Loock B., Grierson D.S., Laville I., Blais J., Doz F., Desjardins L., Carrez D., Guerquin-Kern J.L., Croisy A. (2007). *In vitro* phototoxicity of glycoconjugated porphyrins and chlorins in colorectal adenocarcinoma (HT29) and retinoblastoma (Y79) cell lines. Photodiagn. Photodyn Ther..

[33] Ricchelli F. (1995). Photophysical properties of porphyrins in biological membranes. J. Photochem. Photobiol. B Biol..

[34] Mehraban N., Freeman H.S. (2015). Developments in PDT sensitizers for increased selectivity and singlet oxygen production. Materials.

[35] Debele T.A., Peng S., Tsai H. (2015). Drug carrier for photodynamic cancer therapy. Int. J. Mol. Sci..

[36] Pereira P.M.R., Korsak B., Sarmento B., Schneider R.J., Fernandes R., Tomé J.P.C. (2015). Antibodies armed with photosensitizers: From chemical synthesis to photobiological applications. Org. Biomol. Chem..

[37] Rothemund P. (1936). A new porphyrin synthesis. The synthesis of porphin. J. Am. Chem. Soc..

[38] Adler A.D., Longo F.R., Finarelli J.D., Goldmacher J., Assour J., Korsakoff L. (1967). A simplified synthesis for meso-tetraphenylporphin. J. Org. Chem..

[39] Seybold P., Gouterman P. (1969). Porphyrins XIII: Fluorescence spectra and quantum yields. J. Mol. Spectrosc..

[40] Queiroz K.C.S., Zambuzzi W.F., de Souza A.C.S., da Silva R.A., Machado D., Justo G.Z., Carvalho H.F., Peppelenbosch M.P., Ferreira C.V. (2007). A possible anti-proliferative and anti-metastatic effect of irradiated riboflavin in solid tumours. Cancer Lett..

[41] Insińska-Rak M., Sikorski M. (2014). Riboflavin interactions with oxygen—A survey from the photochemical perspective. Chem. Eur. J..

[42] Mosman T. (1983). Rapid colorimetric assay for cellular growth and survival: Application to proliferation and cytotoxicity assays. J. Immunol. Methods.

