# Supplementary material for: Photophysical Characterization and in Vitro Phototoxicity Evaluation of 5,10,15,20-Tetra(quinolin-2-yl)porphyrin as a Potential Sensitizer for Photodynamic Therapy
Source: Molecules. 2016 Mar 31;21(4):439. doi: 10.3390/molecules21040439 (PMC6273532; doi:10.3390/molecules21040439)
Supplement: Supplementary file 1 [file molecules-21-00439-s001.pdf]

Supplementary Materials: Photophysical Characterization and *in Vitro* Phototoxicity Evaluation of 5,10,15,20-Tetra(quinolin-2-yl)porphyrin as a Potential Sensitizer for Photodynamic Therapy

Letícia D. Costa, Joana de A. e Silva, Sofia M. Fonseca, Cláudia T. Arranja, Ana M. Urbano, Abílio J.F.N. Sobral

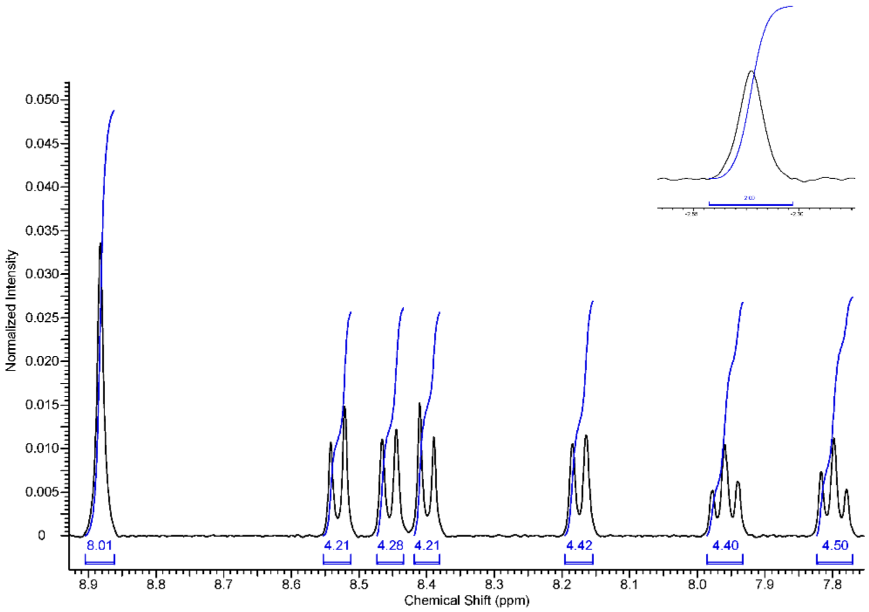

Figure S1. ^1H-NMR spectrum of 2-TQP in $CDCl_3$ at 400.13 MHz.

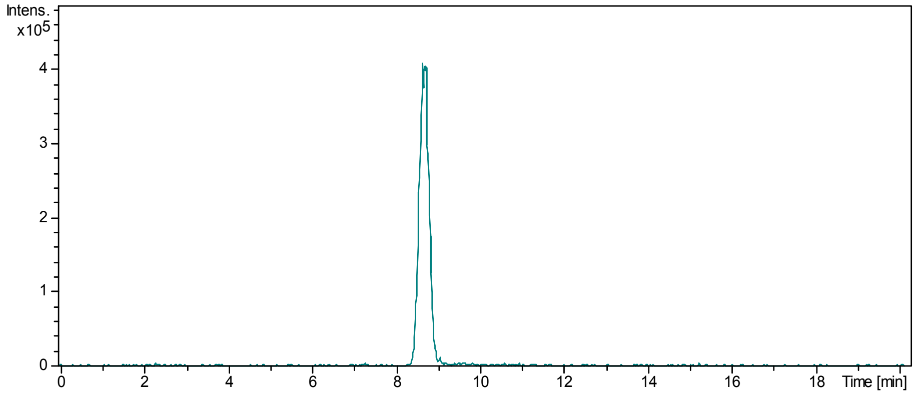

Figure S2. HPLC chromatogram of 2-TQP. The HPLC was carried out with a reverse phase Hichrom 5 C18 (150 × 4.6 mm) column, using a gradient of acetonitrile and formic acid-water (0.1% (*v*/*v*) of formic acid) programmed as follows: 0–5 min, 60% acetonitrile; 5–7 min, 70% acetonitrile; 7–10 min, 80% acetonitrile; 10–20 min, 90% acetonitrile and 100% acetonitrile afterwards. The mobile phase was delivered at a flow rate of 0.80 mL/min.

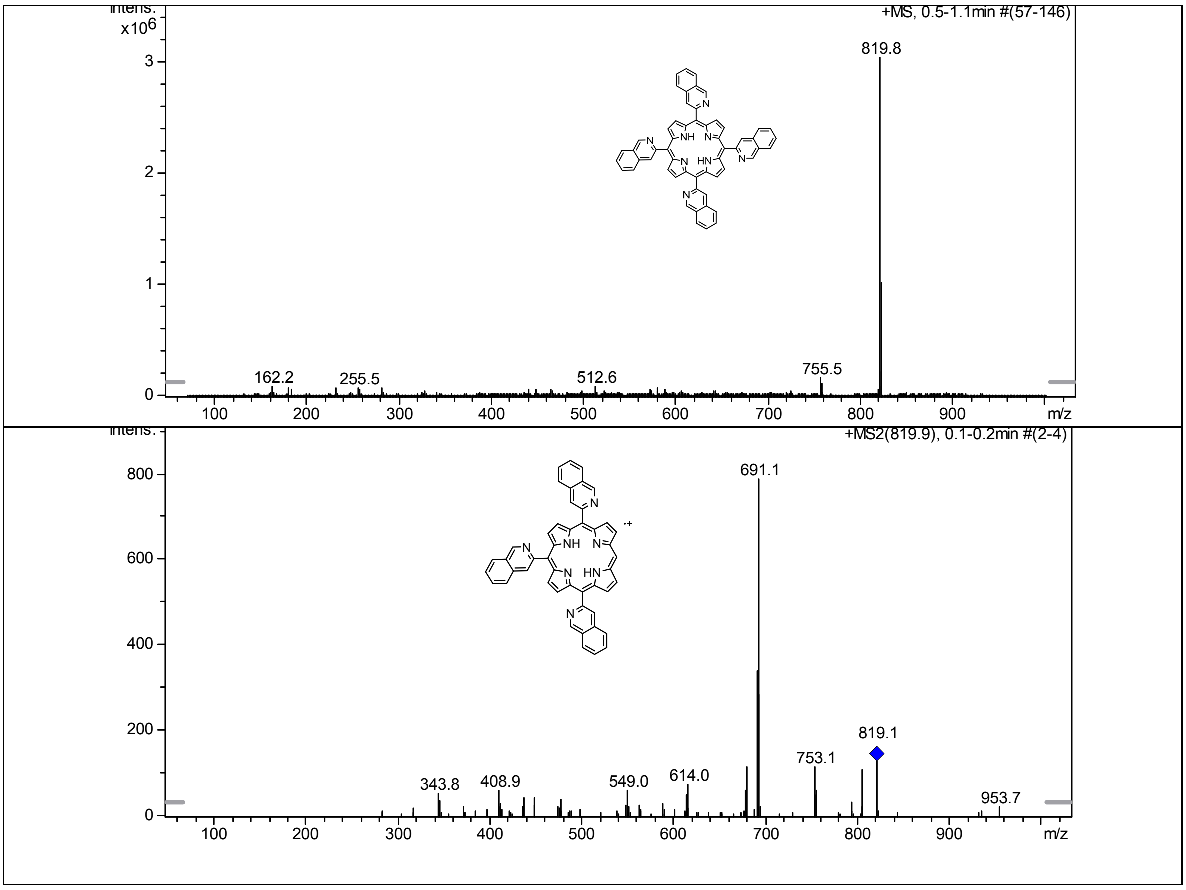

Figure S3. MS and MS/MS spectra of 2-TQP in dichloromethane, clarifying the fragmentation pattern. As expected, the *meso*-quinolone group was lost while no fragmentation of the porphyrin macrocycle was observed.

Calculation of the Octanol-Water Partition Coefficient, miLog P

Hydrophobicity was evaluated through the determination of the octanol-water partition coefficient, log P. The miLogP was calculated using Molinspiration WebME Editor 3.81. The parameters for drug-likeness were evaluated according to the Lipinski's "rule-of-five", using the Molinspiration WebME Editor (http://www.molinspiration.com). As expected, 2-TQP exhibits a miLog *p* value (9.79) similar to that of TPP (9.64). On the other hand, 5,10,15,20-tetra(4-pyridyl)porphyrin (TetraPy) has a miLog *p* value quite different from that of 2-TQP, which means that these two compounds have very different lipophilicities, preventing direct comparisons between their biological activities.

Table S1. Comparison of the drug-likeness property/Lipinski's "rule of five" parameters calculated for 2-TQP and for the already clinically used Foscan®.

Compound	Molecular weight	miLog*P*	*n*-ROTB	*n*-O/N	*n*-OH/NH	*n*-Violations	Volume	TPSA
2-TQP	818.94	9.79	4	8	2	2	721.48	108.93
TPP	614.75	9.64	4	4	2	2	562.14	57.37
TetraPy	618.70	6.99	4	8	2	2	545.51	108.93
Foscan®	680.76	9.07	4	8	6	3	600.39	138.28

n-ROTB, number of rotatable bonds; *n*-O/N, number of hydrogen acceptors; *n*-OH/NH, number of hydrogen bond donors; TPSA, topological polar surface area; *n*-violations, number of violations according to the Lipinski "rule of five".
